# A numerical approach to investigating the mechanisms behind tonotopy in the bush-cricket inner-ear

**DOI:** 10.3389/finsc.2022.957385

**Published:** 2022-08-15

**Authors:** Emine Celiker, Charlie Woodrow, Natasha Mhatre, Fernando Montealegre-Z

**Affiliations:** ^1^ University of Lincoln, School of Life and Environmental Sciences, Joseph Banks Laboratories, Lincoln, United Kingdom; ^2^ Department of Biology, Western University, London, ON, Canada; ^3^ Brain and Mind Institute, Western University, London, ON, Canada

**Keywords:** insect hearing, *crista acustica*, frequency mapping, numerical modelling, bioacoustics, acoustic vibration transmission

## Abstract

Bush-crickets (or katydids) have sophisticated and ultrasonic ears located in the tibia of their forelegs, with a working mechanism analogous to the mammalian auditory system. Their inner-ears are endowed with an easily accessible hearing organ, the *crista acustica* (CA), possessing a spatial organisation that allows for different frequencies to be processed at specific graded locations within the structure. Similar to the basilar membrane in the mammalian ear, the CA contains mechanosensory receptors which are activated through the frequency dependent displacement of the CA. While this tonotopical arrangement is generally attributed to the gradual stiffness and mass changes along the hearing organ, the mechanisms behind it have not been analysed in detail. In this study, we take a numerical approach to investigate this mechanism in the *Copiphora gorgonensis* ear. In addition, we propose and test the effect of the different vibration transmission mechanisms on the displacement of the CA. The investigation was carried out by conducting finite-element analysis on a three-dimensional, idealised geometry of the *C. gorgonensis* inner-ear, which was based on precise measurements. The numerical results suggested that *(i)* even the mildest assumptions about stiffness and mass gradients allow for tonotopy to emerge, and *(ii)* the loading area and location for the transmission of the acoustic vibrations play a major role in the formation of tonotopy.

## 1 Introduction

Tonotopic organisation, or frequency maps, arise in the auditory systems of many species (1). As is well known, the main purpose of this phenomenon is to facilitate frequency analysis of the acoustic vibrations entering the hearing chamber. First discovered in the mammalian ear (2), a tonotopic hearing organ has recently also been observed in the ears of bush-crickets (3–5)], which have been shown to have a hearing system analogous to mammals (6). For bush-crickets, whose ears are located in their forelegs, a non-invasive investigation of the biomechanism governing the inner-ear processes has been possible through the transparent cuticle some species are endowed with (4). In contrast, the structure and location of the mammalian inner-ear makes it experimentally challenging to investigate this mechanism non-invasively (7, 8). Hence, the convergent evolution between the bush-cricket and the mammalian ears provides a unique opportunity to enhance our understanding of these analogous hearing systems, making the investigation of the mechanism behind the workings of the bush-cricket inner-ear a timely and worthy pursuit.

Through his Nobel prize winning work, Georg von Békésy showed that the fluid-immersed basilar membrane in the mammalian inner-ear (the cochlea) acted as a biological Fourier transform, performing frequency analysis on the mechanical travelling wave (2, 9). As the travelling wave moved down the organ, it was observed that the cochlear hair cells (or auditory sensory cells) lying along the membrane would receive mechanical input at specific frequencies, due to an amplitude maxima response dependent on the stiffness and mass gradients of the basilar membrane (10). Some properties of the basilar membrane leading to such gradients include a tapering in width, and a gradual increase in thickness and elasticity (1). Since then, travelling waves have also been observed directly in the basilar papilla of birds (11), and indirectly *via* the timing of responses of auditory-nerve fibres in the auditory organs of some reptiles and frogs (12, 13). Analogous travelling waves have also been measured invasively and non-invasively in the ears of bush-crickets (3, 4, 14, 15). The underlying mechanism is likely more ancient since it has been observed in grigs, suggesting it was shared by a common ancestor (16).

**Figure 1:**
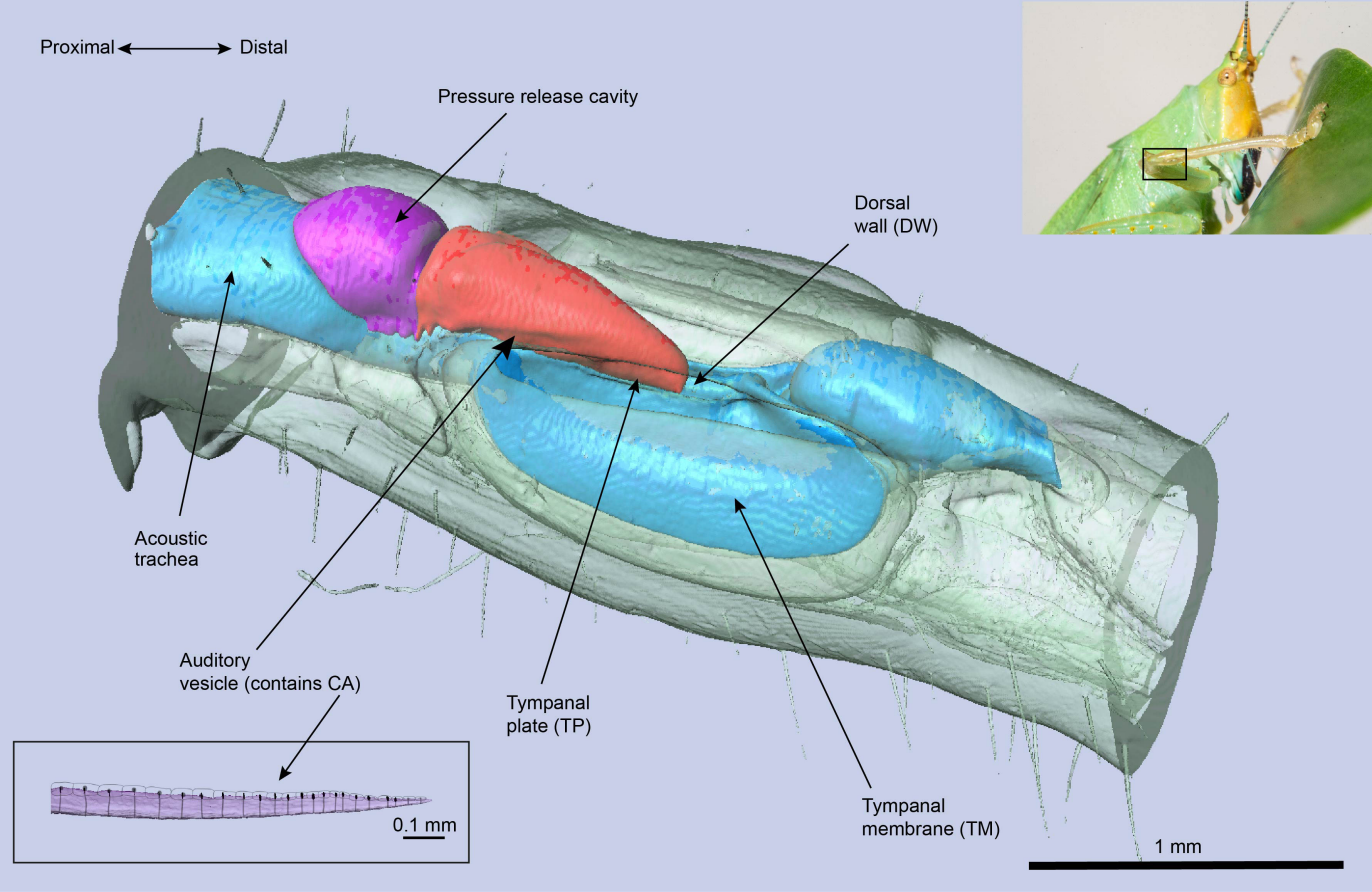
The *μ*-CT image of the *Copiphora gorgonensis* ear and its components.

Bush-crickets have ultrasonic ears that are located in the tibia of their forelegs, and each ear is endowed with two tympanic membranes (TMs). The TMs are backed by an air-filled tube (the acoustic trachea or ear canal), and their outer-ear allows for sound to be received on both sides of the tympana: directly to the external side, and through the acoustic trachea to the internal side (17). The acoustic trachea bifurcates near the TM, with the anterior tracheal branch backing the anterior tympanic membrane, and similarly the posterior tympanic branch lies behind the posterior tympanic membrane. After travelling through the tracheal tube, sound arrives at the TM with a phase difference and a pressure differential compared to the external input (17), leading to the TM acting as a pressure-time difference receiver (18). Acoustic vibrations are then transmitted to the bush-cricket inner-ear, which is a separate, fluid-filled chamber above the TM end of the tracheal tube (see **Figure 1**). The mechanosensory organ of the bush-cricket, the *crista acustica* (CA), is located inside this chamber and lies on the dorsal wall of the anterior tracheal branch. While in other insect hearing systems such as locusts and moths the mechanoreceptors are in direct contact with the TMs (19), this is not the case for bush-crickets, demonstrating another likeness to the mammalian ear (20, 21).

The CA has been observed to resemble an uncoiled basilar membrane (4, 22). Similar to mammals, in the bush-cricket inner-ear the transmitted acoustic vibrations activated the hearing organ, leading to the formation of travelling waves moving along this structure (6). These waves were observed to travel from the narrowest part (distal part) of the sensory organ (the CA) containing the high frequency sensory cells, to the broader (proximal) region containing the low frequency cells (3, 9, 22). As the travelling wave moved along the hearing organ, the CA maximum displacement was seen to occur in a frequency-dependent fashion, after which the wave dissipated, showing clear characteristics of a tonotopically ordered structure (4, 6). Consequently, the frequency dependent movement of the CA activated the relevant mechanosensory cells. Even though the tonotopy observed in the bush-cricket ear is generally attributed to the stiffness and mass gradients of the CA (3), a more detailed analysis, combining experimental and numerical approaches, is required for more conclusive evidence on the mechanism behind this phenomenon.

As is well known, for tonotopical vibrations to emerge along the hearing organ, first the acoustic vibrations have to be transmitted into this chamber through a mechanical phenomenon. For the mammalian ear, the chain of transmission by which sound is captured by the eardrum and delivered, *via* the ossicles to the inner ear is well-studied (23, 24). However, in bush-cricket auditory mechanics, this process is more controversial. To understand the workings of the bush-cricket auditory system, it becomes important to discern the process of vibration transmission into the bush-cricket inner-ear. There are two main arguments in relation to the air-to-liquid conversion of acoustic vibrations in the bush-cricket ear, which are based on a lever-like system (25), where a higher output force is generated through mechanical advantage (26).

The first mechanism was proposed by Bangert et al. (27) by using the species *Polysarcus denticauda* and *Tettigonia viridissima* as model systems, who likened the impedance conversion in the bush-cricket ear to the TM acting as a type 2 lever. A type 2 lever is described as having a fulcrum located at one end with the force applied at the other end. The resulting force is then sensed at the middle of the lever (26). Hence, according to Bangert et al. (27), the force load was sensed at the area of intersection between the TM and the dorsal wall, transferring the airborne acoustic energy to the fluid medium. It was also suggested that there was a contribution to this mechanism from the sound pressure acting on the dorsal wall, emanating from the acoustic trachea (27). A similar mechanism was also put forward by Nowotny et al. (28) for the species *Mecopoda elongata*. Palghat Udayashankar et al. (22), however, proposed that the dorsal wall played a more central role in activating the CA in the *M. elongata* ear, through the pressure of the sound wave on the dorsal wall as it travelled in the acoustic trachea. Hence, it was suggested that the CA obtained a displacement magnitude proportional to its local resonance while stimulated through a pressure parallel to the hearing organ, rather than a travelling wave. A similar idea was also considered for the mammalian inner-ear (29).

A second transmission model was proposed by Montealegre-Z et al. (6), using the species *Copiphora gorgonensis* as a model system. For the neotropical bush-cricket *C. gorgonensis*, the CA is located in the auditory vesicle (see **Figure 1**) which is believed to be derived from the hemolymph channel and is filled with fluid, also bathing the CA [(6) (25)]. The proposed transmission mechanism in (6) was centered around the tympanal plate (TP), a cuticular patch attached to the TM in contact with the distal end of the auditory vesicle (25). The TP was recorded to have an out-of-phase response to its hosting TM, hence performing an air-to-liquid impedance conversion by transmitting the vibrations from the air backed TM to the fluid-filled auditory vesicle. It is suggested that the TP governs this transmission process by acting as a type 1 lever: a lever with the fulcrum in the middle, force applied at one end and the resulting force in the other end.

Therefore, while the role of TPs are not considered by Bangert et al. (27), Montealegre-Z and colleagues (6) suggested that TPs play a central role in the impedance conversion of acoustic vibrations to the fluid medium. Hence, in (6) [see also (25)], the TPs were posited to have an analogous function to the mammalian middle-ear. However, a middle-ear or the auditory vesicle were not observed in the ears of the bush-cricket *M. elongata* (22). There is also a considerable difference in the load areas between the species *M. elongala* and *C. gorgonensis*. For both the species a part of the TM (22, 5) or TP (6) is in contact with the inner-ear fluid, however, the TM has contact along the length of the CA (28), whereas the TP is in contact with only the distal end of the auditory vesicle (6). Thus, in addition to a different lever system, the mechanisms suggested also propose differing sizes of load areas to the inner-ear. While these different models were proposed, it has never been tested whether such mechanical configurations actually give rise to the observed tonotopic behaviour.

**Figure 2:**
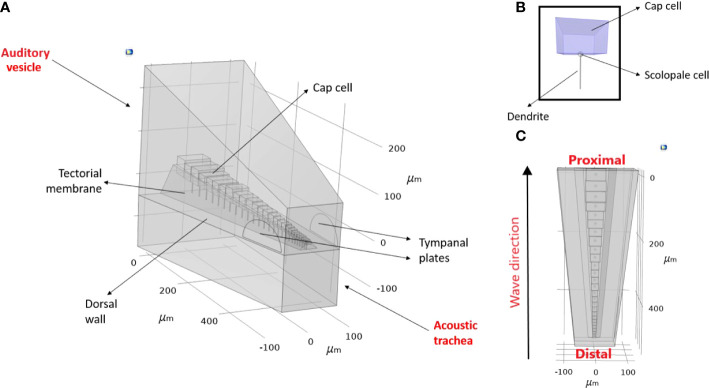
An idealised geometry of (*Copiphora gorgonensis* inner-ear. **(A)** The geometry parameters **(B)** A mechanosensory cell and its components **(C)** The travelling wave direction observed in the bush-cricket inner-ear.

In this study, using *C. gorgonensis* as our model system we investigated the underlying mechanism of the bush-cricket inner-ear. By incorporating simple observable properties, such as mass gradient and tapering (width and height) in the geometry of the CA morphology, we tested mechanical features that are crucial to the development of tonotopy. We also used the constructed models to numerically investigate the effect of the transmission load of acoustic vibrations to the formation of tonotopy. Based on micro-computed tomography (*μ*-CT) measurements of the *C. gorgonensis* inner-ear an idealised geometry was constructed, on which numerical simulations were carried out by manipulating the “middle-ear” conditions. **Figure 2** demonstrates the idealised auditory vesicle and CA geometry. We used this 3D model to test the hypothesis that for *C. gorgonensis*, the existence of a separate chamber, the auditory vesicle, makes it necessary to have a load area with a smaller region as offered by the TP. Further, the role of the dorsal wall in sound transmission was investigated. A comparison with the experimental results in the literature suggested that our numerical results gave the closest match to experimental data when the impedance conversion took place with the influence of both the TP and the dorsal wall.

## 2 Materials and methods

### 2.1 Model geometry

#### 2.1.1 Morphological measurements

To produce an idealised geometry of the *C. gorgonensis* inner-ear, a female specimen was scanned using *(i)* a SkyScan 1172 X-ray *μ* -CT scanner (Bruker Corporation, Billerica, MA, USA) with a resolution of 1.6 *μ* m (40 kV source voltage, 165 *μ*A source current, 2200 ms exposure and 0.1° rotation steps) and *(ii)* synchrotron X-ray CT imaging at the Diamond Manchester Imaging Branchline (I13-2, Diamond Light Source, Oxford). We used monochromatic light and a 4× objective with a pco.edge 5.5 detector, providing a voxel size of 0.8125 *μ*m. The obtained images were then reconstructed with NRecon (v.1.6.9.18, Bruker Corporation, Billerica, MA, USA) for a series of orthogonal slices.

The 3D segmentation of the inner-ear was performed with the software Amira-Aviso 6.7 (Thermo Fisher Scientific, Waltham, Massachusetts, USA), and were used for obtaining the dorsal wall thickness and the dimensions of the auditory vesicle, through the Center Line Tree module in AMIRA. For the 2D measurement of cap cell surface area, scolopale cell radius and dendrite length, an Alicona InfiniteFocus microscope (G5, Bruker Alicona Imaging, Graz, Austria) at ×5 objective magnification was used, with a resolution of about 100 nm.

#### 2.1.2 Idealised geometry

**Figure 3:**
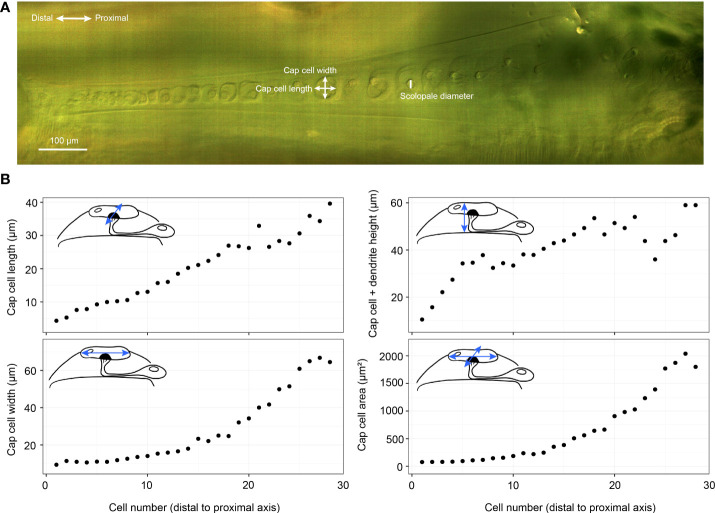
*Crista acustica* (CA) measurements. **(A)** an Alicona InfiniteFocus microscope image of *Copiphora gorgonensis* CA. **(B)** The measurements of *Copiphora gorgonensis* CA components using the Alicona InfiniteFocus microscope. Cap cell area refers to the surface area of the cap cell top surface.

The idealised geometry was constructed in the *Geometry* node of COMSOL Multiphysics, v. 5.6 (30), with parameter dimensions based on the measurements obtained as described in Section 2.1.1. The actual shape of the geometry can be seen in **Figure 1** and **Figure 3**. The acquired geometry is given in **Figure 2**, and the used dimensions are presented in **Figure 3** and **Table 1**. The auditory vesicle itself was represented as an oblong hexahedron. The geometry included 28 individual cap cells and corresponding dendrites of varying dimensions (see **Table 1**), located inside the auditory vesicle. For cap cell geometry, we assumed that they were shaped as cuboids (see **Figure 2B**). Conjoined to the cap cells from the bottom were the scolopale cells which were modelled as spheres, and were also attached to the dendrite. On the other end, it was assumed that dendrites were directly connected to the dorsal wall. The cap cells, scolopale cells and dendrites made up the structure of the modelled CA. The CA was covered by a surface representing the tectorial membrane. Near the distal end of the constructed CA, the TP was modelled to intersect with the auditory vesicle wall. A second geometry with the TM intersecting the acoustic trachea along the length of the CA was also constructed for comparison (see Supplementary Materials, **Figure S1**). A block, intersecting at the dorsal wall, was added representing the acoustic trachea.

**Table 1:** The parameter dimensions applied in constructing the idealised geometry.

Parameter	Dimensions
Auditory vesicle volume	2.22×10^7^ *μ*m^3^
Dorsal wall thickness	Varying linearly in the interval
	3.25-6.5 µm (proximal to distal)
Auditory vesicle wall thickness	15 *μ*m
Cap cell dimensions	Largest 64×40×40 µm^3^ (proximal),
	Smallest 4×9×4 µm^3^ (distal)
Scolopale cell radius	9 *μ*m
Tectorial membrane thickness	2 *μ*m
Dendrite length	59-11 *μ*m (proximal to distal)
Tympanal plate area	5493 *μ*m^2^

### 2.2 Mathematical model

The mathematical models were developed using the Acoustics and Structural Mechanics modules of COMSOL Multiphysics (v. 5.6) (30), and were set-up as an acoustic-structure interaction problem. All the calculations were carried out in the frequency domain so that the system of equations was not time dependent. The auditory vesicle wall and the tectorial membrane were represented with a shell formulation (31), and were coupled with the fluid inside the auditory vesicle using the arbitrary Lagrangian-Eulerian method (32). The fluid was assumed to be water, and the pressure in the fluid was represented by the solution to the linearised Navier-Stokes equations (33). Hence, any possible viscous effects due to the fluid were accounted for. The CA components (cap cells, scolopale cells and dendrites) were assumed to be linear elastic and were represented by the elastic Helmholtz equation (32). The CA was in turn coupled with the fluid inside the auditory vesicle, as well as the auditory vesicle boundary (the shell formulation). In addition, we modelled the propagation of sound in the block representing the acoustic trachea. The block was assumed to be filled with air, and the sound pressure was represented by the solution to the acoustic Helmholtz equation (34). The block was also coupled with the dorsal wall to reflect the influence of the sound pressure in the auditory vesicle due to the propagation of sound in the trachea.

The boundary of the auditory vesicle facing the proximal end of the CA was assumed to act as a pressure release facilitator (6) (see Supplementary Materials, **Figure S2**), as a result of a *Free boundary condition* (34) defined there. This condition ensures that the boundary moves based on its material properties and applied forces, and is not constrained except at the edges. A free boundary condition was also defined on the dorsal wall. The remainder of the auditory vesicle walls were fixed. At the proximal end face of the idealised acoustic trachea, the sound wave travelling through the tracheal tube was modelled as a harmonic wave of frequency *f* and amplitude 1 Pa (see Supplementary Materials, **Figure S2**). On this face, we also defined a *radiation boundary condition* so that there were no reflections there (34). The transmission of vibrations through the TP were represented with an *acceleration* condition with a magnitude of *ω* ^2^ *μ*m/s^2^, where *ω* = 2*π f* is the angular frequency (34). The frequency was considered in the interval 10-90 kHz with a resolution of 10 kHz.

**Table 2** summarises the material properties employed in the mathematical models. These values were selected through parametric sweeps, as a result of giving the closest numerical results to the experimental data (see Supplementary Materials, Section 1 for more details). The material properties were assumed to be isotropic and homogeneous.

**Table 2:** The parameter material properties applied in the mathematical models.

Parameter	Young’s Modulus
Dorsal wall	0.5 GPa
Auditory vesicle wall	1 GPa
Cap cell, scolopale cell	50 MPa
Dendrite	1 GPa
Tectorial membrane	10 MPa
Tympanal plate	1 GPa
Tympanic membrane	1 GPa

The model described above was also adapted to represent the condition of a TM transmission of acoustic vibrations to the auditory vesicle rather than a TP transmission, where a larger area along the length of the auditory vesicle received the force. This was achieved by a manipulation of the idealised geometry (see Supplementary Materials, **Figure S1**). TP and TM entrances without the influence of the dorsal wall were also considered by removing the idealised acoustic trachea from the geometry.

### 2.3 Numerical simulations

The variational form of the developed mathematical models were solved using the finite-element method. Linear Lagrange elements were used for the solution so that a second order accuracy was obtained in the *L* ^2^–norm (35). For the constructed finite-element mesh, the tetrahedral mesh radii were between 1-20 *μ*m. This mesh-size ensured that there were at least 10 tetrahedral elements per wavelength for the considered frequency range of 10-90 kHz. The mesh size was also based on the thickness of the potential viscous boundary layers forming near the boundaries. From the linearized Navier-Stokes equations, the thickness of the viscous boundary layer can be obtained as


$$\delta =\sqrt{\frac{2\mu }{\omega \rho_{0}}},$$

where *μ* = dynamic viscosity, *ω* = 2*π f* is the angular frequency with *f* frequency, and *ρ*_0_ the background density (36). It is clear that the thickness *δ* depends on the properties of the fluid considered, and that it becomes thinner with the increase of frequency. Since the auditory vesicle is assumed to be filled with water, at 20°C and 90 kHz the viscous layer thickness *δ* = 1.8853 *μ*m. Hence, the adopted mesh size allows for capturing the solution even in the thinnest boundary layer.

A mesh stability analysis is presented in Supplementary Materials, Section 3. The COMSOL Multiphysics (30) inbuilt feature of mixed interpolation of tensorial components (MITC) shell elements were used for the meshing of the auditory vesicle boundary. MITC shell elements have been established as effectively capturing different shell behaviours with varied and complex stress conditions (37). The obtained mesh was a conforming finite-element mesh. The constructed mesh is demonstrated in Supplementary Materials, **Figure S3**.

## 3 Results

In this study, we undertook a numerical investigation of the mechanism behind the workings of the *C. gorgonensis* inner-ear, the auditory vesicle. We found that a mass gradient generated by the changing size of the cap cells, and the geometry of the CA and dorsal wall were sufficient to generate tonotopic vibrations. In addition, the two main hypotheses of vibration transmission, *(i)* through the TP and dorsal wall, and *(ii)* through the TM and dorsal wall, were tested numerically to determine their influence on the formation of tonotopy. To investigate the individual contributions of the dorsal wall, TP and TM, their effects on the system were also simulated separately.

Throughout this section, distance measurements refer to the distance from the proximal end, as defined in **Figure 2C**.

### 3.1 Combined methods of sound transmission

**Figure 4:**
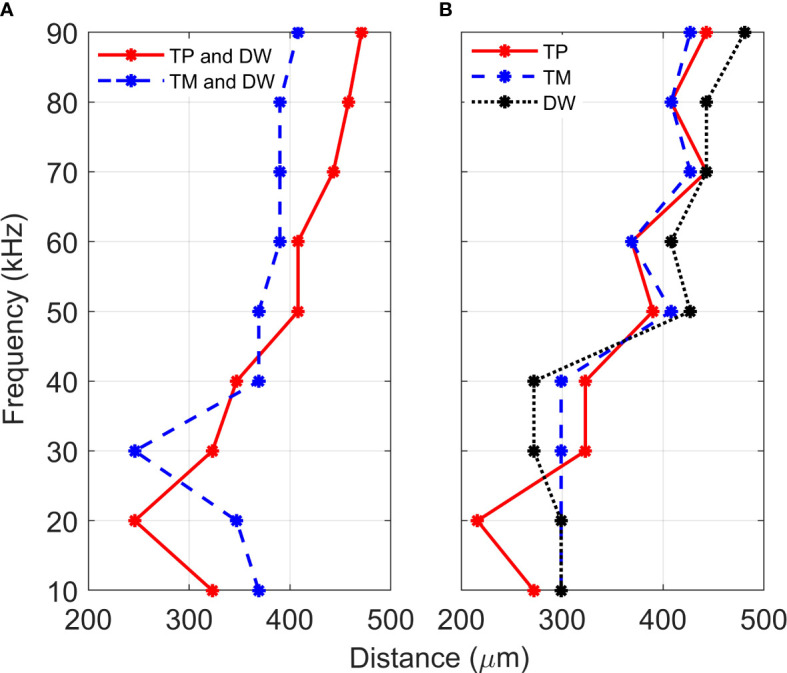
Location of displacement maxima. Distance, from the proximal end, of the vertical displacement maxima of *crista acustica* when vibrations are transmitted **(A)** through the tympanal plate and dorsal wall (TP and DW) or the tympanic membrane and dorsal wall (TP and DW), **(B)** through the tympanal plate (TP), tympanic membrane (TM) or the dorsal wall (DW).

The mathematical models were set-up to represent a combined method of sound transmission: through the *(i)* TP and dorsal wall, and the *(ii)* TM and dorsal wall. For an analysis of tonotopy along the CA, we checked for monotonicity in the relationship between frequency and distance, in the range of frequencies and positions sampled. **Figure 4** demonstrates the results obtained for vertical displacement maxima location along the CA, for vibrations in the frequency range 10-90 kHz. As can be observed, the tonotopical arrangement, or a frequency dependent displacement maxima, was most pronounced when the TP and dorsal wall transmissions were considered together.

**Figure 5:**
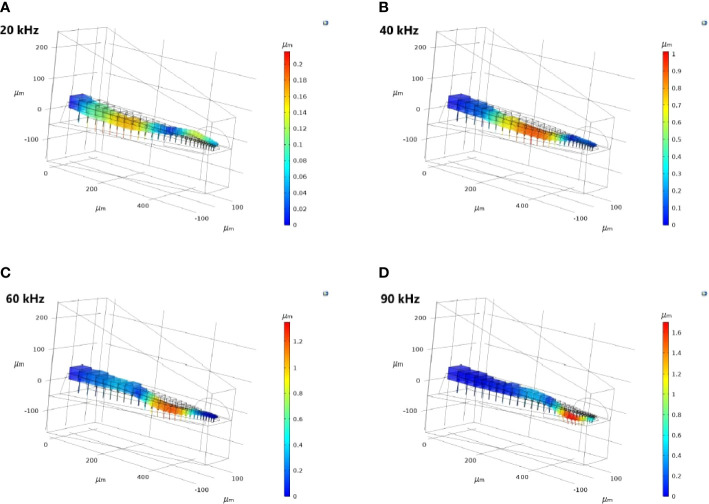
The three-dimensional *crista acustica* (CA) vertical displacement facilitated by the tympanal plate (TP) and dorsal wall (DW) transmission of acoustic vibrations at **(A)** 20 kHz, **(B)** 40 kHz, **(C)** 60 kHz, **(D)** 90 kHz.

**Figure 6:**
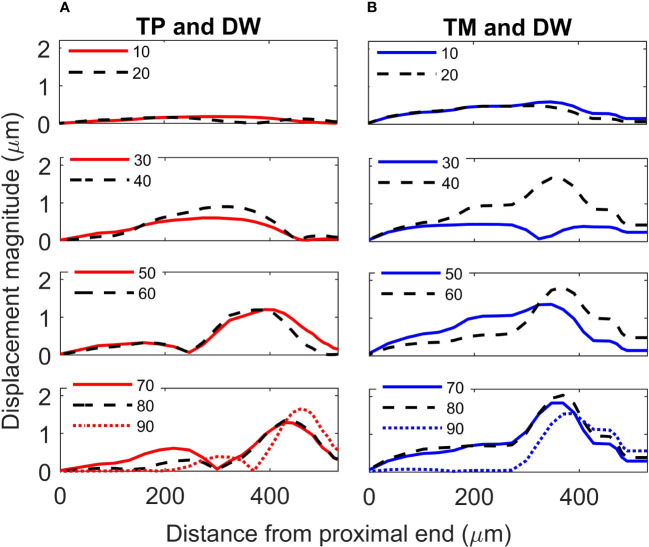
*Crista acustica* vertical displacement magnitude due to transmission of acoustic vibrations at various frequencies. Displacement magnitude due to transmission of vibrations facilitated by the **(A)** tympanal plate and dorsal wall (TP and DW) shown in the left column, **(B)** tympanic membrane and dorsal wall (TM and DW) shown in the right column.

For the TP and dorsal wall input, the three-dimensional image of CA vertical displacement at various frequencies is presented in **Figure 5**. For this input system, an increase in frequency in the interval 20-90 kHz lead to the movement of the displacement maxima location from 246 *μ*m to 471 *μ*m (see **Figure 4A**). However, between 10 kHz and 20 kHz a discontinuity was observed in the tonotopic vibrations, and the displacement maxima location was at 408 *μ*m for both 50 kHz and 60 kHz. The displacement magnitude with respect to distance is given in **Figure 6A**. The results demonstrate a non-decreasing displacement with the increase of frequency. From **Figure 5** and **Figure 6A** it can also be observed that the displacement maxima are smooth peaks, dissipating a short distance from the maxima.

A tonotopical pattern of vibration was not observed when both the TM and dorsal wall served as vibrational inputs to the CA (see **Figure 4A** and Supplementary Materials, **Figure S4**). In the frequency range 30-90 kHz, the displacement maxima lied within the short interval 369 - 408 *μ*m from the proximal end, showing the spatial frequency gradient to be less differentiated. Further, displacement maxima at 30, 40 kHz were located at 360 *μ*m, and similarly for 60-80 kHz they were located at 390 *μ*m. Tonotopy was also not present in the frequency range 10-30 kHz. The distance versus vertical displacement at the CA, for the TM and dorsal wall input is given in **Figure 6B**. A correlation between frequency and displacement magnitudes was not present.

### 3.2 Single method of sound transmission

To determine the individual effects of the dorsal wall, TP and TM input on the development of tonotopic vibrations along the CA, the mathematical models were solved with these three parameters as the separate excitation methods of the system. **Figure 4B** demonstrates the displacement maxima location along the CA with respect to vibration frequency. As can be observed from **Figure 4B**, when these input methods are considered separately, there is a lack of monotonicity between frequency and distance. More precisely, some displacement maxima locations were obtained as:

*(i)* For TP input 390 *μ*m at 50 kHz; 369 *μ*m at 60 kHz; 443 *μ*m at 70 kHz; 408 *μ*m at 80 kHz;

*(ii)* For DW input 299 *μ*m at 20 kHz; 272 *μ*m at 40 kHz; 427 *μ*m at 50 kHz; 408 *μ*m at 60 kHz; 443 *μ*m at 70 kHz;

*(iii)* For TM input 299 *μ*m at 40 kHz; 408 *μ*m at 50 kHz; 369 *μ*m at 60 kHz; 427 *μ*m at 70 kHz; 408 *μ*m at 80 kHz;

While a TP input leads to displacement maxima closest to tonotopy, a fluctuation of the displacement location is present throughout the considered frequency range. These fluctuations become smaller in magnitude as the frequency increases. Similar fluctuations also resulted from TM and dorsal wall inputs in the frequency range 40-90 kHz. For the dorsal wall and TM excitations a correlation between frequency and displacement maxima location can not be discerned in the range 10-40 kHz.

The displacement magnitude along the CA obtained through the three inputs is given in Supplementary Materials, **Figure S5**. The dorsal wall excitation led to displacement magnitudes that were orders of magnitude smaller than those generated by the TP or TM excitation of the system.

## 4 Discussion

In this study, we numerically investigated the mechanism behind the formation of tonotopical vibrations along the CA. Further, we analysed how the transmission load area of acoustic vibrations influenced this formation in the inner-ear.

### 4.1 Factors sufficient for developing tonotopy

The local resonance frequency (*f*) of an acoustic structure can be determined by the ratio of its stiffness (*s*) to its mass (*m*) in the form


(1) $$f=\frac{1}{2\pi }\sqrt{\frac{s}{m}},$$

where the stiffness is dependent on the elasticity (Young’s modulus) and the dimensions of the structure, and mass is the product of the structure’s density and volume. From equation (1) it is easy to see that a change in stiffness or mass leads to the change of the resonant frequency of the structure.

Due to a decrease in width and increase in thickness and elasticity from the base to the apex, stiffness and mass gradients are also present along the mammalian basilar membrane (9). As a result, these stiffness and mass gradients contribute to the formation of the observed tonotopical vibrations there (12). Using a combined experimental and numerical approach, Olson and Nowotny (14) demonstrated that the bush-cricket CA also had a similar stiffness magnitude to the basilar membrane, and that the stiffness decreased from the high-frequency region to the low-frequency region. Nevertheless, when such a structure is located inside a complex system, there are several factors that can modify the local behaviour. In particular, for the bush-cricket inner-ear these changes can arise from the oscillators in the system being coupled to each other, the standing wave cavity resonance of the auditory vesicle, and the standing wave resonances of the TM and the dorsal wall. Hence, while equation (1) is generally true for a single degree of freedom, it may not hold when the degrees of freedom increase. Applying a three-dimensional numerical analysis, we aimed to determine the extent to which the CA and dorsal wall geometry contributed to the emergence of the experimentally observed tonotopy there.

To incorporate the dimensions of the CA into the models, we used the precise *μ* –CT measurements of *C. gorgonensis* inner-ear parts (see **Figure 3**). While it was not possible to directly use the scanned geometry in our models due to the difficulties of forming a finite-element mesh on the detailed features of the CA, we constructed an idealised geometry based on the obtained measurements (**Figure 2**). As a result, the numerical models contained a CA tapering in width and height. A similar property was also present for the dorsal wall, due to the narrowing of the auditory vesicle towards the distal end of the chamber. Hence, the model included the geometrical factors considered to be necessary for the frequency dependent displacement of the CA. The elasticity of these parts, however, were assumed to be homogeneous and isotropic in the simulations. In addition, the geometry did not include the curvature present along the bush-cricket dorsal wall and CA, which could potentially add another gradient through its effect on the stiffness (38).

Since the geometry of the TP and dorsal wall input system matches the *C. gorgonensis* ear morphology, and TP input is the mechanism identified for vibration transmission to the *C. gorgonensis* inner-ear experimentally (6), in this section we refer to this set of data when talking about our results. This data set demonstrates a frequency based displacement along the CA (see **Figure 4A**). Further, the maximum displacement peak is a smooth peak dissipating a short distance after passing through the point of resonance (**Figure 6**), a tonotopical property also observed experimentally (6). Hence, we can see that tonotopy emerge as a result of changes in the CA and dorsal wall geometry that produce simplified stiffness and mass gradients.

Nevertheless, a discontinuity can be observed in the tonotopic organisation around 10 kHz (see **Figure 4**), implying the requirement of additional features to the model for more definitive tonotopy. Such a discontinuity implies that certain properties have more significant effects at smaller frequencies, for instance anisotropic elasticity, curvature or damping properties. Another discrepancy between the experimental data [(3) (6)] and our results can also be seen at the tonotopy placement. While Montealegre-Z et al. (6) recorded the displacement maxima for 30 kHz at a distance of about 200 *μ*m from the proximal end, for the numerical results it was located at a distance of 320 *μ*m, showing a shift in tonotopy at the numerical results. Based on formula (1), a shift in the maxima location points at a difference in stiffness and mass between the actual and numerical geometry, which once again suggests the requirement of more realistic material and geometric properties in the model. Hence, for the investigation of these discrepancies with the experimental data, as well as for the further understanding of the inner-ear mechanism, an enhancement of the model is certainly worth pursuing. Still, it is worth noting that the distances presented in (6) were measured through the insect cuticle rather than directly on the CA, which was the case with the numerical results.

We believe our numerical approach exhibits the utility of employing three-dimensional numerical models for investigating a complex system alongside an experimental approach. For the bush-cricket hearing system, while there are many studies successfully explaining the general workings of the inner-ear [(3, 6, 14, 25, 27) and references therein], it is not experimentally possible to pick inner-ear parts apart to determine their individual functions, without compromising the underlying mechanism of the system to a certain extent. Our numerical technique provides an alternative approach to such an analysis, which can be a powerful tool in obtaining reliable predictions on the inner-ear mechanism. Our approach is a first attempt to represent the system with a simpler structure in order to understand the contribution of specific properties, namely the basic morphology of the chamber and its components.

Another simplifying assumption we applied for setting up the mathematical models was that the TM and TP were comprised of homogeneous and isotropic materials. Through experimental investigations it has been observed that the tympana boost frequencies relevant for the communication of conspecific bush-crickets (5), indicating a more complex material structure. In the mathematical models, properties like mass, stiffness and damping of the TM and the TP, essential for capturing the impedance of the system were also not based on measured values. Hence, the magnitude of the displacement maxima presented in **Figure 6** and Supplementary Materials, **Figure S5** are not necessarily a true reflection of the displacement magnitude. While the model predictions are not directly comparable to the observed data, the constructed model provides a simplified adaptation of the biomechanics of the inner-ear, from which it is still possible to obtain qualitative information related to tonotopy. Primarily the model suggests that it is possible to develop a tonotopic vibrational map, based on the mass and spatial gradients that result purely from geometrical changes.

### 4.2 Differentiation in CA morphology and the role of TP, TM and DW inputs

In the mammalian hearing system, the transmission of acoustic vibrations into the inner-ear is well-studied [see (23) (24) (39), and references therein]. In particular, the middle ear is comprised of three tiny bones (the ossicles) which through a lever action pass the eardrum vibrations to the fluid filled cochlea, performing an air-to-liquid impedance conversion (39). For bush-crickets, however, there are still multiple untested ideas in the literature with regards to the sound transmission to the bush-cricket inner-ear. Nevertheless, the two main propositions for transmission mechanisms are both based on lever systems. For instance, for the species *M. elongata*, it is believed that the whole TM acts as the main input for sound transmission (3) (5), through functioning as a type 2 lever. Hence, the *M. elongata* ear will receive the maximum load at the intersection of the TM with the dorsal wall. It has been observed that a large section of the TM is in contact with the air-filled acoustic trachea, and a smaller section is attached to the fluid-filled hemolymph channel, thus allowing for impedance conversion. For *M. elongata*, The TM is in contact with the hemolymph channel along the length of the CA (28). In contrast, for *C. gorgonensis*, vibration transmission was identified to be facilitated by the type 1 lever action of the TP (6). Thus, the large deflections of the airborne TM are transformed into smaller deflections of the fluid-bound TP, demonstrating clear impedance conversion characteristics in the ear. Moreover, the TP is observed to be in contact with the fluid only towards the distal end of the CA. Our numerical results suggest that the load area, or the dimensions and location of the TM or TP in contact with the inner-ear, also play a significant role in the formation of tonotopy inside the inner-ear.

As can be observed from **Figure 5**, the frequency dependent change in displacement maxima along the CA is evident when the vibrations enter through the TP, with the maxima moving from the proximal end to the distal end of the CA as frequency increases. This is less pronounced for a TM entrance to the *C. gorgonensis* inner-ear (Supplementary Materials, **Figure S4**). Nonetheless, some differences between the bush-cricket *C. gorgonensis* [established TP input (6)] and *M. elongata* [established TM input (5, 28)] are worth pointing out. The species *C. gorgonensis* have a separate inner-ear chamber, the auditory vesicle. Whereas for *M. elongata*, the CA is located in the hemolymph channel, hence the mechanics driving the formation of tonotopy may be different. In our numerical simulations, the geometry only reflected the dimensions of the *C. gorgonensis* auditory vesicle. Interestingly, Bangert et al. noted that for the species *Polysarcus denticauda* and *Tettigonia viridissima*, while the CA was activated through the TM, the tympana was in contact with the hemolymph channel only where the high frequency receptor cells of the middle and distal crista acustica were located (27). In this study, the outer surface of the TM for the considered species were identified to have an area called the ‘inner plate’, which was a dark oval and stiff area that was surrounded by a paler area, assumed to be more elastic. However, no out-of-phase response between the inner-plate and the hosting tympana was recorded, and the tympana was identified as a uniformly vibrating membrane. Hence, there may be greater variation in auditory mechanics even within bush-crickets than previously assumed. Accordingly, we appreciate the differences in morphologies between bush-cricket species and from this point on limit our conjectures to the species *C. gorgonensis*.

The model also elucidates the transmission properties of the TM, TP, and the role of the dorsal wall which has also been a topic of interest [(22, 27)]. While vibrational input from the wall was not reported in (6), the influence of sound pressure from the dorsal wall was suggested in (5) and (27). In (22), it was further proposed that the dorsal wall activated the CA before vibrations even reached the TM. To analyse the dorsal wall influence numerically, we simulated the transmission of sound by removing the acoustic trachea from underneath the dorsal wall in the geometry, and activated the system only through the TM or the TP (see **Figure 4B**). In addition, we also considered a simulation where the transmission was only through the dorsal wall. As can be observed in **Figure 4**, the removal of the dorsal wall pressure for a TP and TM excitation did not lead to a tonotopical arrangement, highlighting the role of the wall displacement itself on the movements of the CA. A solitary dorsal wall (**Figure 4**) excitation of the system did not lead to the experimentally observed tonotopy at the CA either. Hence, we conjecture that in addition to the effect of the stretching and squeezing of the dorsal wall through the TM or the TP entrance, the sound pressure from the acoustic trachea below also plays a significant role in activating the mechanoreceptors. However, the small displacement magnitude from the dorsal wall input (Supplementary Materials, **Figure S5C**) leads us to believe that a stronger mechanism than a solitary dorsal wall stimulation is needed for the excitation of the system. These results suggest that the resonance approach proposed in (22) is not a probable mechanism for the stimulation of the *C. gorgonensis* CA.

Our attempt to simulate the biomechanical processes in the bush-cricket ear, using a 3-dimensional idealised geometry, has resulted in a reasonable qualitative match to the experimental results in the literature. This suggests that for the investigation of such processes, a numerical approach can provide a cost efficient alternative and validation method to empirical studies. Reliable numerical models, validated through experimental data, also provide a new platform for analysing the individual effects of the parameters comprising the bush-cricket inner-ear, which is not possible through experimentation. Therefore, the further numerical investigation of the bush-cricket inner-ear, through improving the incorporated quantitative (material properties, fluid viscosity, potential non-Newtonian fluid properties) and geometrical (curvature of the dorsal wall and CA) properties is certainly worth pursuing.

## Data availability statement

The datasets presented in this study can be found in online repositories. The names of the repository/repositories and accession number(s) can be found below: https://drive.google.com/file/d/1xC7xfYp9896oEItITREDaAfGn5zLx-ZK/view?usp=sharing.

## Author contributions

EC, NM and FM-Z contributed to conception and design of study. CW took morphological measurements. EC developed numerical models, ran simulations and analyzed obtained results. EC and CW developed figures and images. EC wrote the first draft of the manuscript. All authors contributed to manuscript revision, read, and approved the submitted version.

## Funding

EC, CW and FM-Z are funded by the European Research Council Grant ERCCoG-2017-773067 (to FMZ for the project “The Insect Cochlea”). NM is funded by an NSERC Discovery grant and supplement (687216, 675248) and Canada Research Chair (693206).

## Acknowledgments

We thank the Orthopterists’ Society for aiding the funding of the micro-CT work of CW, and the University of Lincoln for CW’s PhD studentship.

## Conflict of interest

The authors declare that the research was conducted in the absence of any commercial or financial relationships that could be construed as a potential conflict of interest.

## Publisher’s note

All claims expressed in this article are solely those of the authors and do not necessarily represent those of their affiliated organizations, or those of the publisher, the editors and the reviewers. Any product that may be evaluated in this article, or claim that may be made by its manufacturer, is not guaranteed or endorsed by the publisher.
