# Supplementary material for: A numerical approach to investigating the mechanisms behind tonotopy in the bush-cricket inner-ear
Source: Front Insect Sci. 2022 Aug 15;2:957385. doi: 10.3389/finsc.2022.957385 (PMC10926389; doi:10.3389/finsc.2022.957385)
Supplement: Supplementary file 1 [file DataSheet_1.pdf]

Supplementary Material

1 MODEL MATERIAL PROPERTIES

The Young's modulus of the *Copiphora gorgonensis* inner-ear components were determined through parametric sweeps. We carried out these sweeps using the values given in Table S1 and combinations there of. The values presented in Table S1 are within the known bounds of insect cuticle [1]. The selection criteria of the final values to be employed in the simulations were based on the displacement magnitude of the *crista acustica*, which was expected to have a tonotopical pattern of vibration.

Table S1. Young's modulus values tested using parametric sweeps for the components of the idealised bush-cricket inner-ear

Parameter	Young's modulus (in GPa)
Dorsal wall	0.05, 0.1, 0.5, 1, 5
Auditory vesicle wall	1, 5, 10, 15, 20
Scolopale cell	0.05, 0.1, 0.5, 1, 5
Dendrite	0.05, 0.1, 0.5, 1, 5
Tectorial membrane	0.01, 0.05, 0.1, 0.5, 1, 5
Tympanal plate	1, 5, 10, 15, 20
Tympanic membrane	1, 5, 10, 15, 20

2 FIGURES

1. Figure S1 demonstrates the idealised geometry of the bush-cricket *C. gorgonensis* inner-ear, the auditory vesicle. The geometry has been manipulated to reflect an assumed tympanic membrane (in blue) transmission of the acoustic vibrations into the inner-ear. The tympanic membranes in the geometry intersect the auditory vesicle throughout the length of the hearing organ *crista acustica*, as observed in different bush-cricket species.

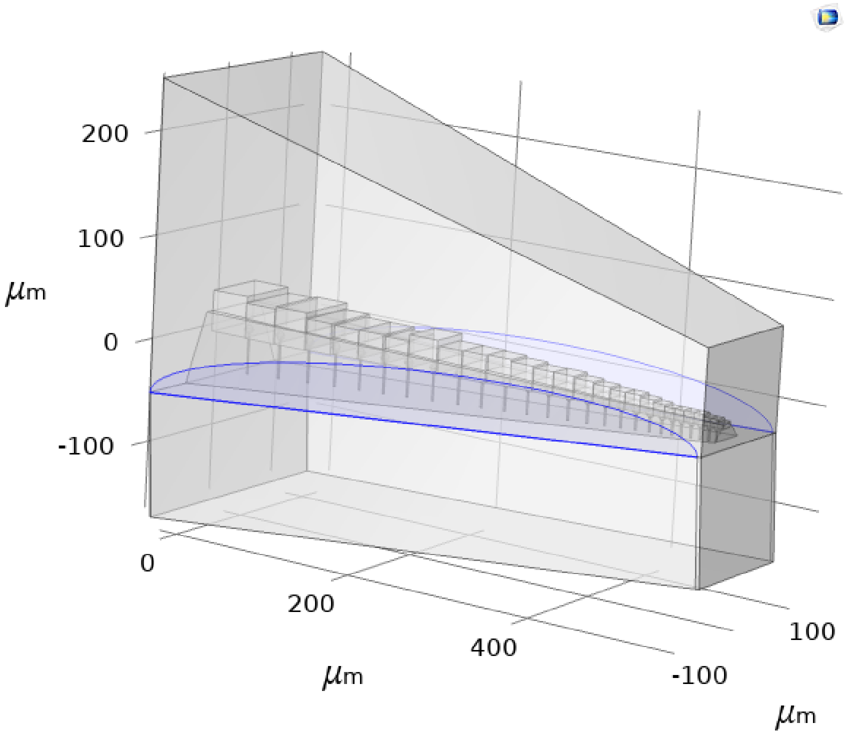

Figure S1. Idealised geometry with a tympanic membrane transmission into the auditory vesicle.

2. For the set-up of the mathematical model, the wall at the proximal end of the geometry was assumed to be a pressure release facilitator, hence no restrictions were placed on this wall (a free boundary condition). Similarly, no restrictions were placed on the movements of the dorsal wall either. The remainder of the walls were assumed to be fixed, so that there was no movement there. At the artificial acoustic trachea, the boundary at the proximal end is where the sound pressure entrance is defined. The selections are given in Figure S2.

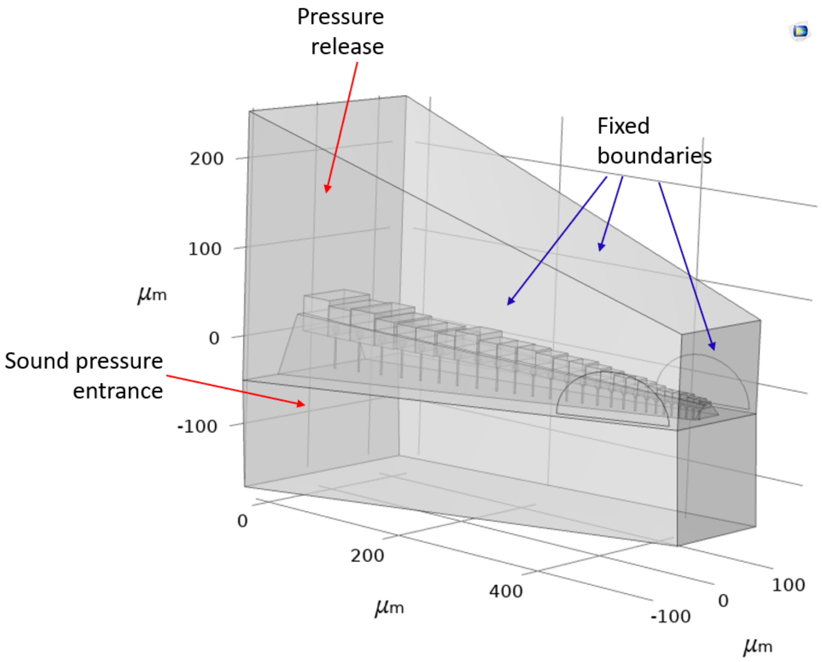

Figure S2. Conditions specified on the idealised auditory vesicle boundary.

3. The finalised finite-element mesh is demonstrated in Figure S3.

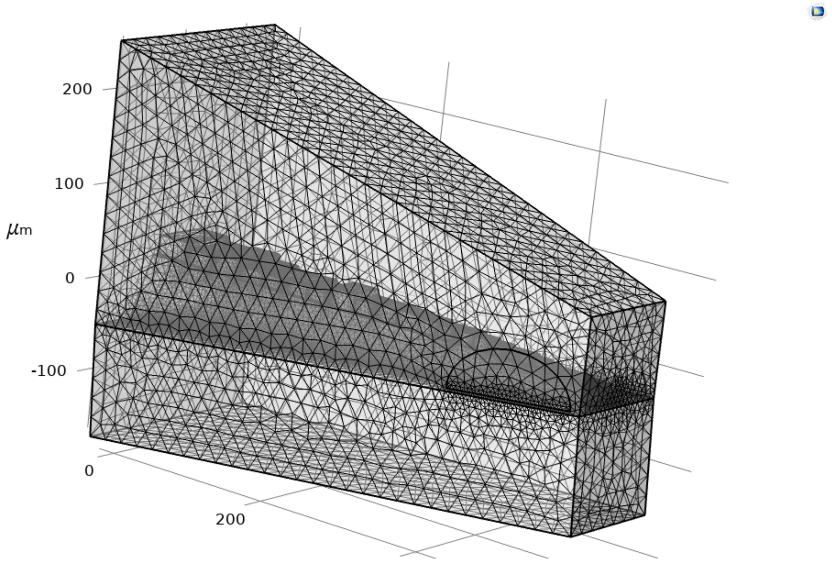

Figure S3. The finite-element mesh formed in the idealised geometry of the *C. gorgonensis* ear.

4. Figure S4 demonstrates the vertical displacement magnitudes of the *crista acustica* as simulated using the geometry given in Figure S1. A change in the frequency of the transmitted vibrations does not lead to a tonotopical displacement.

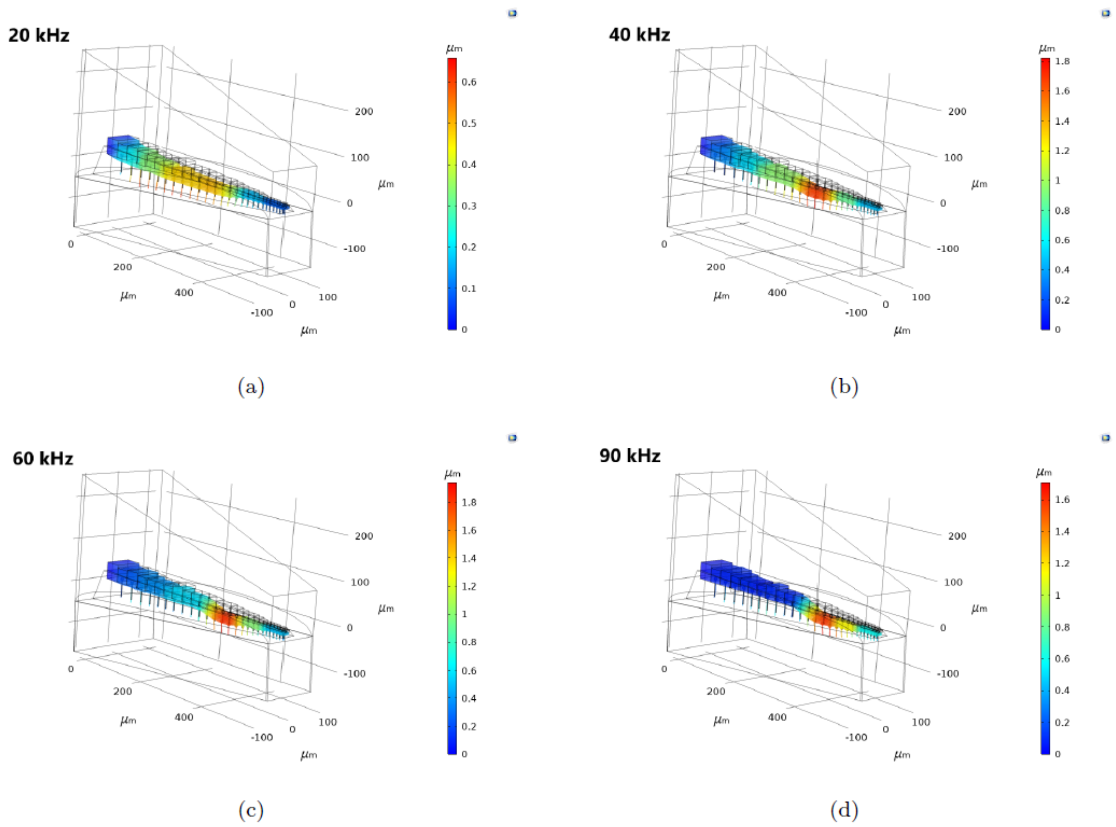

Figure S4. The three-dimensional *crista acustica* (CA) vertical displacement facilitated by the tympanic membrane (TM) and dorsal wall (DW) transmission of acoustic vibrations at (a) 20 kHz, (b) 40 kHz, (c) 60 kHz, (d) 90 kHz.

5. The effect of having three different vibration transmission methods on the *crista acustica* displacement is given in Figure S5. The system was excited by three different methods, through the tympanal plate (TP), the tympanic membrane (TM) and the dorsal wall (DW).

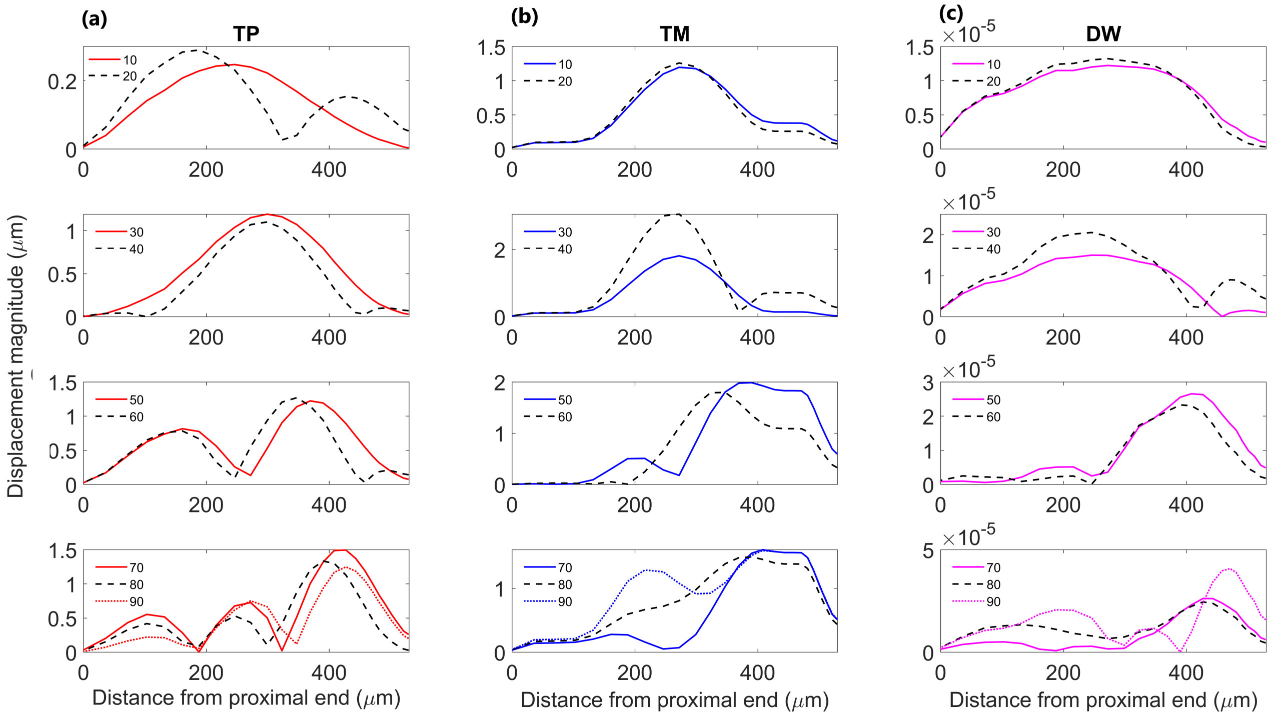

Figure S5. *Crista acustica* (CA) vertical displacement magnitude due to transmission of acoustic vibrations at 10-90 kHz. Displacement magnitude of vibrations facilitated by transmission through (a) tympanal plate (TP) given in the left column, (b) tympanic membrane (TM) given in the middle column, and (c) dorsal wall (DW) given in the right column.

3 MESH SENSITIVITY ANALYSIS

To test for the mesh dependence of the finite-element results, the solution was obtained on three different mesh sizes with the following number of tetrahedral elements:
(i) Mesh 1 = 796261 (element radii 1.5-25 μm),
(ii) Mesh 2 = 1060973 (element radii 1-20 μm),
(iii) Mesh 3 = 1219814 (element radii 0.5-15 μm).
The convergence of the solution was considered at fixed points in the domain, where the mesh reduction error was calculated by looking at the difference of the solutions from two consecutive mesh sizes, in the maximum norm. The reduction errors presented in Table S2 are the maximum error obtained in the frequency range 10-90 kHz. A graphical representation of the mesh effects is also given in Figure S6, at the two extreme frequencies considered. Both Table S2 and Figure S6 demonstrate that the solution is independent of the mesh size.

Table S2. The maximum convergence error between consecutive meshes

Mesh	Convergence error
Mesh 1 & 2	0.1132
Mesh 2 & 3	0.0407

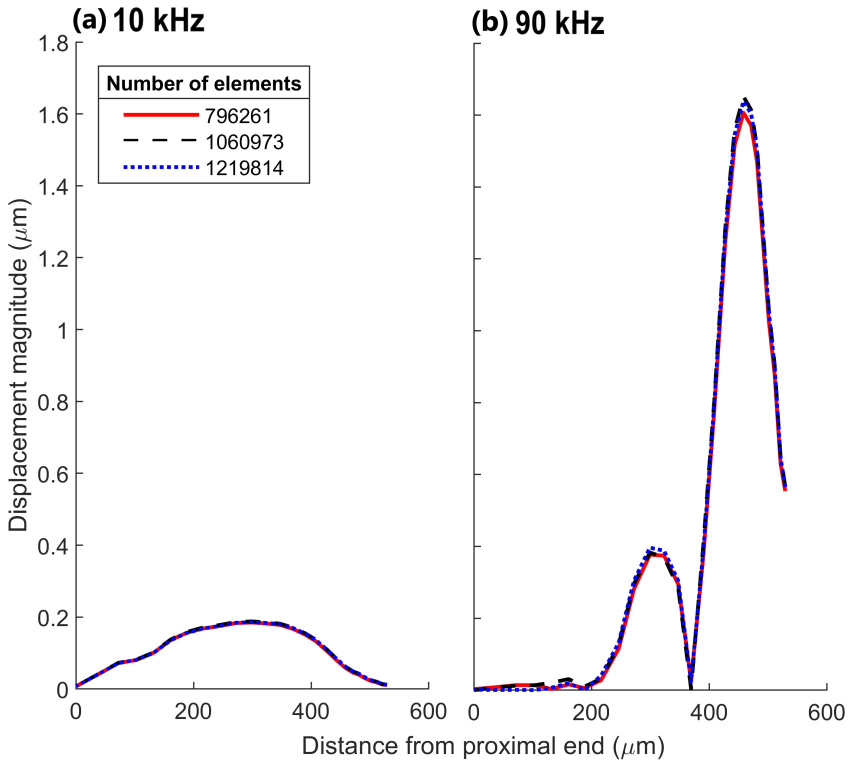

Figure S6. The maximum displacement obtained along the *crista acustica* at frequencies (a) 10 kHz and (b) 90 kHz. The solution is obtained on three different finite-element meshes of increasing refinement.

REFERENCES

1. Vincent, J. F., & Wegst, U. G. (2004). Design and mechanical properties of insect cuticle. *Arthropod structure & development*, **33**(3), 187-199.
